# Sodium Valproate Use in Japanese Patients with Schizophrenia and Coronavirus Disease Is Associated with an Increased Risk of Pneumonia

**DOI:** 10.3390/jcm12185953

**Published:** 2023-09-13

**Authors:** Yusuke Arai, Daimei Sasayama, Akira Kuraishi, Reiko Sahara, Shiho Murata, Akira Tanaka, Kotaro Amemiya, Nobuteru Usuda, Kazuaki Kuraishi, Shinsuke Washizuka

**Affiliations:** 1 Department of Psychiatry, Shinshu University School of Medicine, Matsumoto-City 390-8621, Japan; y-arai@shinshu-u.ac.jp (Y.A.); swashi@shinshu-u.ac.jp (S.W.); 2 Department of Psychiatry, Kurita Hospital, Nagano-City 380-0921, Japan; kuraishi@kuritahp.or.jp (A.K.); sahara@kuritahp.or.jp (R.S.); shiho@view.ocn.ne.jp (S.M.); unabone100g@gmail.com (A.T.); camelparliament@yahoo.co.jp (K.A.); usudakenbikyou@gmail.com (N.U.); kuritahp@avis.ne.jp (K.K.)

**Keywords:** schizophrenia, COVID-19, pneumonia, mood stabilizer, sodium valproate

## Abstract

Schizophrenia is a known risk factor for coronavirus disease (COVID-19) infection and severity, and certain psychotropic drugs have been linked to increased mortality in infected patients with schizophrenia. However, little evidence exists regarding this risk. We retrospectively examined the association between mood stabilizers and the risk of pneumonia in patients with schizophrenia. This study included 99 patients with schizophrenia or schizoaffective disorder who were infected with COVID-19 in 2022 and met the inclusion criteria. After conducting propensity score matching to align patient backgrounds and concomitant medications, we assessed the impact of mood stabilizers, specifically sodium valproate, on the risk of pneumonia development. Univariate analysis revealed that patients with schizophrenia and COVID-19 who developed pneumonia were more likely to be older (64.5 [14.2] vs. 57.4 [11.5] years, *p* = 0.008) and using sodium valproate (44.4% vs. 16.7%, *p* = 0.004). Even after propensity score matching, patients who developed pneumonia were still more likely to be receiving sodium valproate than not (58.8% vs. 20.0%, *p* = 0.003). Sodium valproate use may be a risk factor for the development of pneumonia in patients with chronic schizophrenia who are infected with COVID-19 during long-term hospitalization.

## 1. Introduction

The incidence of coronavirus disease (COVID-19) has been increasing since its emergence, and as of March 2023, over 757 million confirmed cases and 6.8 million deaths have been reported globally [1]. In Japan, despite large-scale vaccination, approximately 5000 new confirmed cases were reported daily from January to February 2022, during which this study was conducted [2]. Schizophrenia is a known risk factor for COVID-19 infection and severity [3]. Factors that may contribute to this include higher rates of comorbidities such as hypertension, diabetes, and chronic lung disease among patients with schizophrenia [4]. In addition to these factors, patients with schizophrenia may be more susceptible to severe COVID-19 disease and could experience higher mortality rates when taking psychiatric medications [5]. Japanese psychiatric hospitals already face problems with discharge rates [6], polypharmacy, and high-dose prescriptions for long-term patients [7]. In this context, preventing COVID-19 clusters in psychiatric hospitals and monitoring drug adjustments when they occur are important issues. Psychotropic drugs, such as antipsychotics and benzodiazepines, have been associated with increased mortality among patients hospitalized for COVID-19 [8,9]. Specifically, in a recent study, exposure to second-generation antipsychotics (SGAs) was associated with an increased 30-day mortality rate and exposure to first-generation antipsychotics (FGAs) with a decreased 30-day discharge rate [10]. For clozapine, an increased risk of severe COVID-19 infection was reported compared to other antipsychotics, although the risk of severe illness during COVID-19 infection has not been evaluated [11]. In the case of benzodiazepines, it was observed that coronavirus-positive patients who were using benzodiazepines faced an elevated risk of hospitalization compared to patients who were not using these medications. Additionally, long-term users of benzodiazepines exhibited a higher risk in this context [12]. However, the relationship between the use of mood stabilizers (MSs), particularly sodium valproate (VPA), and the severity of COVID-19 infection remains uncertain and is the subject of ongoing research. Previous studies have reported that the prevalence of concurrent MS use among schizophrenia patients in Japan was 28.8% [13]. This rate is higher than that observed in other Asian countries, making the association between MS use in schizophrenia patients and the risk of COVID-19 severity a crucial clinical question in Japan. Some studies have suggested the potential for VPA and lithium carbonate (LI) to have a preventive effect against pneumonia caused by COVID-19 [14,15]. In contrast, a previous cohort study of hospitalized patients with severe mental illness revealed a significant increase in mortality associated with the use of VPA [16]. These studies underscore the importance of recognizing differences in patient characteristics, including the type and severity of mental illness. Hence, it is imperative to investigate and analyze the relationship between MS use and the severity of COVID-19 infection within various subgroups categorized by mental health conditions and patient backgrounds. This approach allows for a more nuanced understanding of how mood stabilizers may impact the outcomes of COVID-19 in specific patient populations. In this context, we hypothesized that the use of MSs, especially VPA, in elderly chronic schizophrenia patients could worsen the outcome of COVID-19 infection. To explore this hypothesis, we retrospectively investigated the association between MS use, particularly VPA, and the risk of pneumonia in schizophrenia patients who became infected with COVID-19 during their stay in a Japanese psychiatric hospital.

## 2. Materials and Methods

### 2.1. Ethics Statement

The study protocol was approved by the Institutional Review Board of Kurita Hospital (approval number: 202203). In this study, we used clinical information obtained during routine clinical practice, and informed consent was obtained through an opt-out option, which was promoted on the hospital’s website and bulletin boards. This approach is recommended for epidemiological studies by the Ministry of Health, Labor, and Welfare in Japan [17].

### 2.2. Study Design and Patients

**Figure 1:**
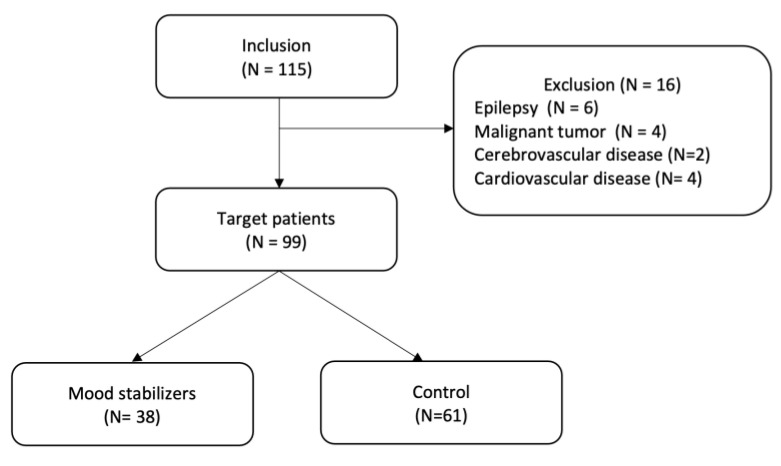
Study flow chart. In total, 115 participants were recruited for this study; 16 patients were excluded because of comorbid epilepsy, malignant tumors, cerebrovascular disease, or cardiovascular disease and 99 patients were included in the analysis.

This retrospective single-center observational study enrolled patients who were part of a COVID-19 cluster that developed in the chronic ward of a psychiatric hospital between January and February 2022. This study included 115 patients diagnosed with schizophrenia or schizoaffective disorder by an experienced psychiatrist, according to the Diagnostic and Statistical Manual of Mental Disorders, Fifth Edition [18]. The inclusion criteria were previous vaccination for COVID-19 and PCR positivity for COVID-19 between 20 January and 19 February 2022. The exclusion criteria were other known risk factors for severe COVID-19: comorbidities with malignancy, cerebrovascular disease, cardiovascular disease, chronic kidney disease, chronic lung disease, liver disease, or immunodeficiency [19]. Furthermore, because we aimed to target MSs for psychiatric symptoms, we excluded patients with epilepsy. After 16 patients were excluded, 99 patients were eligible for analysis. Of these, 38 patients were in the group receiving MSs, and the 61 patients not receiving MSs comprised the control group (Figure 1). This study was conducted in compliance with the STROBE guidelines.

### 2.3. Procedures and Measures

We extracted basic clinical information from the electronic medical records such as sex, age at the time of COVID-19 infection, and any psychotropic drugs that the patient had been taking continuously for one week before the onset of COVID-19. The classifications and definitions of psychotropic drugs were as follows. Antipsychotics were categorized into FGAs and SGAs. MSs included VPA, LI, carbamazepine, and lamotrigine. Sleeping pills and anxiolytics were classified as benzodiazepines, Z-drugs, orexin receptor antagonists, and melatonin receptor agonists. Psychotropic doses were recorded as chlorpromazine equivalents (CP-eq) for antipsychotics [20], diazepam equivalents (DZP-eq) for benzodiazepines and Z-drugs [21], and LI equivalents (LI-eq) for MSs [22]. COVID-19 severity was classified by an internal medicine specialist as mild, moderate, or severe based on the diagnostic criteria [23,24]. Patients with mild severity were categorized as having no pneumonia, while those with moderate or severe severity were categorized as having pneumonia.

### 2.4. Statistical Analysis

**Figure 2:**
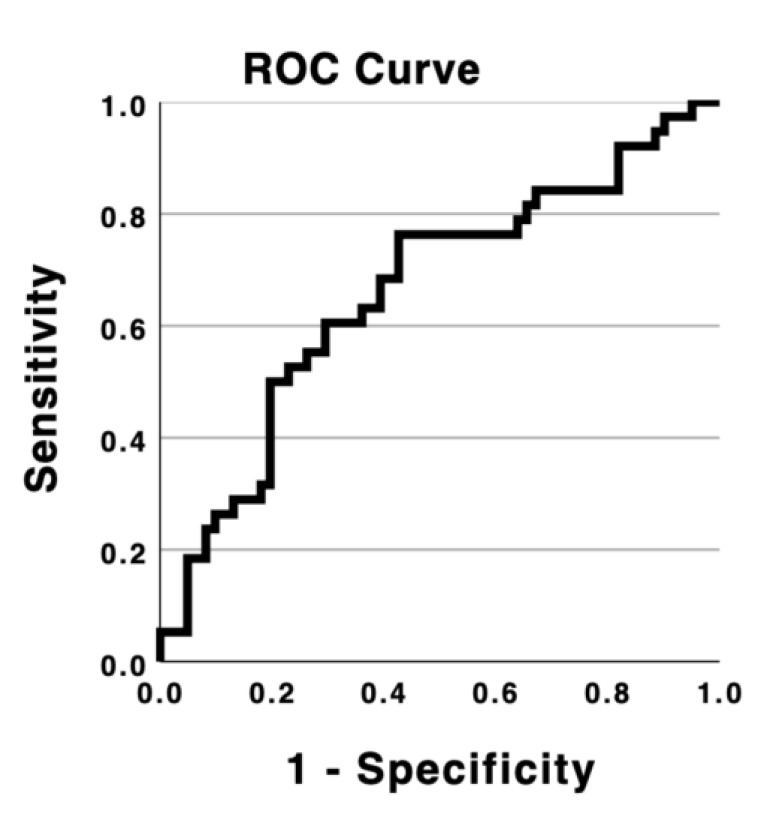
ROC curve of the propensity score matching model (ROC, receiver operating characteristic).

Means and standard deviations were calculated for continuous variables, and frequencies and percentages were calculated for categorical variables. Univariate analysis was performed to compare the patient’s background, comorbidities, medications, and outcomes. The chi-square test was used to compare categorical variables, whereas the Mann–Whitney U test was used to compare continuous variables. The cohort was divided into two groups (those with or without pneumonia), and univariate analysis was performed to evaluate the impact of the usage of MSs, as well as the effect of different subtypes of MS, on pneumonia development in patients with schizophrenia. Subsequently, propensity score matching was performed to control for confounding factors in the analysis of MS use. The propensity score was calculated with logistic regression for estimating the probability that a patient would receive MSs. We defined the following variables as potential confounders: age, sex, CP-eq of antipsychotics, and DZP-eq of benzodiazepines. CP-eq and sex were chosen as independent variables, taking cues from previous research [13], which indicated that individuals receiving higher doses of MSs tended to have higher mean doses of antipsychotic medications, and that patients prescribed MSs were more likely to be female. Additionally, DZP-eq was included as an independent variable under the assumption that MS administration may be correlated with both benzodiazepine use and antipsychotic medication use. Moreover, age was included as an independent variable, with the presumption that clinicians might exhibit a tendency to avoid polypharmacy in elderly patients due to concerns about tolerability. A 1:1 nearest-neighbor risk-set matching algorithm without replacement was used with the propensity score, with a maximum caliper width set at 0.2 of the standard deviation of the logit of the propensity score. Consequently, the statistical analysis included 31 patients from both the MS and control groups. In logistic regression analysis conducted to calculate the propensity score, the area under the receiver operating characteristic (ROC) curve (AUC) for this model was 0.661, with a 95% confidence interval (CI) ranging from 0.549 to 0.774 (Figure 2). Statistical significance was set at *p* < 0.05, and the *p*-values were adjusted for multiple comparisons using the Bonferroni correction. All analyses were performed using the Statistical Package for the Social Sciences version 29 (SPSS; IBM Corp., Armonk, NY, USA).

The purpose of the propensity score matching was to control for confounding factors in the analysis of mood stabilizer use. In the logistic regression analysis used to calculate the propensity score, potential confounders such as age, sex, CP-eq of antipsychotics, and DZP-eq of benzodiazepines were included as covariates. The AUC was 0.661 (95% CI: 0.549–0.774), which quantifies the model’s discriminatory power in predicting the likelihood of treatment assignment. The ROC curve is a graphical representation of the model’s performance in distinguishing between patients who were using mood stabilizers and those who were not based on the propensity score. In this case, an AUC of 0.661 indicates a moderate discriminatory ability of the model in predicting the usage of mood stabilizers in patients with schizophrenia. ROC = receiver operating characteristic; CP-eq = chlorpromazine equivalent; AUC = area under the receiver operating characteristic curve; CI = confidence interval.

## 3. Results

### 3.1. Demographic and Clinical Characteristics

Table 1 presents the demographic and clinical characteristics of the MS and control groups. The mean [standard deviation] age of the MS group was 55.9 (13.7) years, which was significantly younger than that of the control group (61.5 [11.6] years, *p* = 0.038). The MS group had significantly fewer cases of comorbid hypertension than the control group (5.3% versus [vs.] 21.3%, *p* = 0.030). Regarding medications, the MS group had a significantly higher rate of combined use of FGAs and SGAs (44.7% vs. 21.3%, *p* = 0.014) and a significantly higher antipsychotic CP-eq dose than the control group (1020.4 [467.5] vs. 808.6 [514.2], *p* = 0.020). Furthermore, the MS group was significantly more likely to use melatonin receptor agonists than the control group (13.2% vs. 0%, *p* = 0.004). The MS group had a significantly higher incidence of pneumonia than the control group (39.5% vs. 19.7%, *p* = 0.031). Only the difference in the percentage of melatonin receptor agonist use between the MS and control groups met the criterion of *p* < 0.01 after multiple comparison adjustments with the Bonferroni correction.

### 3.2. Risk Factors for the Development of Pneumonia

Table 2 shows the association between each factor and the development of pneumonia. Patients who developed pneumonia were significantly older (64.5 [14.2] vs. 57.4 [11.5] years, *p* = 0.008) and were more likely to be male (59.2% vs. 34.7%, *p* = 0.031). Considering medications, patients who developed pneumonia were more likely to use MSs (55.6% vs. 31.9%, *p* = 0.031) and higher LI-eq doses (347.8 [429.1] vs. 223.9 [449.6], *p* = 0.025). Furthermore, patients who developed pneumonia were more likely to use VPA (44.4% vs. 16.7%, *p* = 0.004). Older age and VPA use were significant (*p* = 0.01) after multiple comparison adjustment criteria with the Bonferroni correction.

### 3.3. Risk Factors for the Development of Pneumonia after Propensity Score Matching

Table 3 presents the demographic and clinical characteristics of the MS and control groups after propensity score matching was conducted. After the matching process, 31 patients were successfully matched using fuzzy matching, but 7 patients remained unmatched because of missing keys. The MS group exhibited a significantly higher incidence of pneumonia than the control group (38.7% vs. 16.1%, *p* = 0.046).

In Table 4, risk factors for the development of pneumonia after propensity score matching are shown. Despite the matching process, patients who developed pneumonia were still more likely to be older (66.9 [14.2] vs. 57.0 [11.3] years, *p* = 0.003) and to be using VPA (58.8% vs. 20.0%, *p* = 0.003).

## 4. Discussion

The propensity score matching in this study strongly suggests that patients with pneumonia exhibited significantly higher rates of VPA usage. Considering these results in the context of our study cohort, we can conclude that VPA may indeed serve as a risk factor for pneumonia in older, chronic patients with schizophrenia who are infected with COVID-19.

This conclusion aligns with the findings of a previous study involving hospitalized patients with serious mental illness, where VPA usage was associated with increased infection and a parallel increase in mortality rates [16]. These results bolster our findings, providing further support for our conclusion. However, it is important to acknowledge that our study results appear to contradict previous research suggesting that VPA has the potential to prevent pneumonia in patients with epilepsy and mental disorders [14]. Furthermore, another previous study investigating the COVID-19 infection rate and severity in VPA users found that VPA users had significantly lower infection rates and hospitalization risks compared with the general population [25]. VPA has a history of being investigated as a treatment for various viral infections. The inhibitory effect of VPA on inflammatory cytokines, such as TNF-α and IL-6, has been considered to prevent the generation of cytokine storms by COVID-19 [26]. Although excessive immune responses may also be involved in the pathophysiology of schizophrenia [27,28], it is not known exactly how the immunosuppressive function of VPA affects schizophrenic patients during COVID-19 infection. On the other hand, a pilot study involving 241 epilepsy patients aimed at determining whether VPA had a preventive effect against COVID-19 infection or severe disease did not support the hypothesis of a preventive effect [29].

To reconcile these differences in results, it is crucial to consider the variations in the patient populations studied. Notably, these differences encompass variations in the type and severity of mental illnesses. In this previous study indicating the effectiveness of VPA in preventing pneumonia, it was generally assumed that the patient populations had bipolar disorder, epilepsy, and other conditions such as neuropathic pain migraines [14]. In contrast, our study, along with previous studies highlighting the association between VPA use and COVID-19, pneumonia, or increased mortality [16], focused on patients hospitalized with schizophrenia. Taken together, the use of VPA may increase the risk of severe COVID-19 outcomes in patients hospitalized with schizophrenia. Additionally, considering that the mean age of our cohort is approximately 60 years, which is older than the populations studied in previous research investigating the association between VPA and COVID-19 severity, it is possible that VPA may have different effects during COVID-19 infection, which are dependent not only on the type and severity of the disease of interest but also on age. In summary, based on the results of our study, we conclude that VPA may indeed represent a risk factor for pneumonia in older, severe, and chronic patients with schizophrenia who are infected with COVID-19. This conclusion considers the complexities of the patient populations studied and aligns with the existing evidence, emphasizing the need for further research to elucidate the intricate relationship between VPA, psychiatric disorders, and COVID-19 outcomes.

Recent research into the relationship between COVID-19 severity and psychotropic medications has consistently highlighted the detrimental impact of antipsychotics and benzodiazepines, especially when used in high doses [8,9,10,12]. This knowledge has significantly contributed to our understanding of COVID-19 risk factors. However, the findings from this study are particularly noteworthy due to the relatively scarce information available on MSs in the context of COVID-19. When considering the collective insights from previous studies and the results presented here, it becomes evident that the immune response of individuals with schizophrenia might be influenced by various factors, including age, the severity of their condition, and the duration of their illness. This nuanced perspective highlights the need for further research to comprehensively understand how these factors interact and impact the susceptibility and outcomes of COVID-19 in individuals with schizophrenia. Such understanding is crucial for tailoring effective clinical interventions and improving the care of this vulnerable population.

This study possesses several strengths and limitations that warrant discussion. The major strength of the present study was that patients shared the same living environment and were infected with COVID-19 at roughly the same time, resulting in a COVID-19 cluster. This shared environment minimizes the impact of potential confounding factors as the patients were infected with the same genotype of SARS-CoV-2 and had similar living conditions.

The limitations of this study are as follows. First, the present study was conducted at a single site, which restricts the generalizability of the results to a broader population. Future research should aim for more diverse and multicenter samples to enhance the external validity of the findings. Second, due to the observational design of the study, causal relationships cannot be established between variables. Third, psychiatric comorbidities, the severity of illness, and socioeconomic disadvantages were not assessed in the present study. The presence of comorbidities may have acted as confounding variables affecting COVID-19 morbidity and mortality [30]. This limitation emphasizes the need for more comprehensive psychiatric assessments in future investigations. Additionally, given that the mean age of our cohort is approximately 60 years, which is generally older than the populations studied in previous research investigating the association between VPA and COVID-19 severity, it is possible that VPA may have different effects during COVID-19 infection, depending not only on the type and severity of the disease of interest but also on age. Forth, the sample size was small, which limits the generalizability of the findings. Due to the retrospective nature of the study, the sample size was determined by the available data without a prior power analysis. Larger, well-powered studies are needed for more robust conclusions. Finally, the sample size was inadequate relative to the number of explanatory variables when applying the logistic regression analysis used for calculating propensity scores. This may have affected the reliability of the analysis.

In summary, while this study offers valuable insights, especially within the unique cluster setting, it is important to acknowledge its limitations. Future research with larger, diverse samples and more rigorous diagnostic assessments is necessary to build upon and contextualize these findings.

## 5. Conclusions

This study is one of the few to investigate the risk of pneumonia in patients with schizophrenia who were infected with COVID-19 while hospitalized in a Japanese psychiatric hospital. The study results suggest that VPA may be a risk factor for severe COVID-19 infection in older patients with chronic schizophrenia. The study offers valuable insights but should be interpreted in the context of its limitations. Future research with larger, diverse samples and more rigorous diagnostic assessments is needed to build upon these findings and enhance their generalizability.

## Figures and Tables

**Table 1:** Demographic and clinical characteristics.

	MSs	Control	*p*-Value
Physical Background			
Age, years	55.9 (13.7)	61.5 (11.6)	0.038 *
Sex, male	16 (42.1%)	27 (44.2%)	0.833
BMI, kg/m^2^	20.3 (3.9)	20.8 (3.9)	0.574
Hospitalization durations, years	7.5 (6.7)	8.2 (8.3)	0.911
Smoker	0	0	-
Comorbidity			
Hypertension	2 (5.3%)	13 (21.3%)	0.030 *
Hyperlipidemia	2 (5.3%)	7 (11.5%)	0.296
Diabetes mellitus	2 (5.3%)	3 (4.9%)	0.939
Medication			
VPA	24 (63.2%)	-	-
LI	8 (21.1%)	-	-
CBZ	10 (26.3%)	-	-
LTG	4 (10.5%)	-	-
LI-eq, mg	681.8 (486.0)	-	-
Antipsychotics	38 (100%)	61 (100%)	-
FGAs	17 (44.7%)	16 (26.2%)	0.058
SGAs	38 (100%)	58 (95.1%)	0.165
FGAs + SGAs	17 (44.7%)	13 (21.3%)	0.014 *
CP-eq, mg	1020.4 (467.5)	808.6 (514.2)	0.020 *
Benzodiazepines	24 (63.2%)	27 (44.2%)	0.067
Z-drugs	5 (13.2%)	7 (11.5%)	0.803
DZP-eq, mg	6.9 (7.4)	5.4 (8.4)	0.085
Orexin receptor antagonists	10 (26.3%)	13 (21.3%)	0.566
Melatonin receptor agonists	5 (13.2%)	0	0.004 *
Antidepressant	3 (7.9%)	3 (4.9%)	0.546
Outcome			
Pneumonia	15 (39.5%)	12 (19.7%)	0.031 *
Death	0	0	-

* *p*-value < 0.05; continuous variables are presented as mean (SD); categorical variables are presented as n (%); univariate analysis was performed for each factor between patients who were prescribed mood stabilizers and those who were not. Abbreviations: BMI = body mass index; MSs = mood stabilizers; VPA = sodium valproate; LI = lithium carbonate; CBZ = carbamazepine; LTG = lamotrigine; eq = equivalent; FGAs = first generation antipsychotics; SGAs = second generation antipsychotics; CP = chlorpromazine; DZP = diazepam; SD = standard deviation.

**Table 2:** Risk factors for the development of pneumonia.

	Pneumonia	No Pneumonia	
Variable	(N = 27)	(N = 72)	*p*-Value
Physical Background			
Age, years	64.5 (14.2)	57.4 (11.5)	0.008 *
Sex, male	16 (59.2)	25 (34.7)	0.031 *
BMI, kg/cm^2^	21.0 (3.2)	20.5 (4.1)	0.474
Comorbidity			
HT, HL, and/or DM	8 (29.6%)	18 (25.0%)	0.641
Medication			
Mood Stabilizers			
Users	15 (55.6%)	23 (31.9%)	0.031 *
LI-eq, mg	347.8 (429.1)	223.9 (449.6)	0.025 *
VPA Users	12 (44.4%)	12 (16.7%)	0.004 *
LI Users	2 (7.4%)	6 (8.3%)	0.880
CBZ Users	3 (11.1%)	7 (9.7%)	0.838
LTG Users	2 (7.4%)	2 (2.8%)	0.298
Antipsychotic			
Users	27 (100%)	72 (100%)	-
CP-eq, mg	854.7 (554.5)	903.1 (488.7)	0.511
Benzodiazepine			
Users	16 (59.2%)	35 (48.6%)	0.345
DZP-eq, mg	6.3(6.8)	5.8 (8.5)	0.394

* *p*-value < 0.05; continuous variables are presented as mean (SD); categorical variables are presented as n (%); univariate analysis was performed for each factor between those who developed pneumonia and those who did not. Abbreviations: BMI = body mass index; HT = hypertension; HL = hyperlipidemia; DM = diabetes mellitus; VPA = sodium valproate; LI = lithium carbonate; CBZ = carbamazepine; LTG = lamotrigine; eq = equivalent; CP = chlorpromazine; DZP = diazepam; SD = standard deviation.

**Table 3:** Demographic and clinical characteristics after propensity score matching.

	MSs	Control	*p*-Value
Variable	N = 31	N = 31	
Physical Background			
Age, years	59.2 (12.3)	60.3 (11.4)	0.637
Sex, male	15 (48.4%)	13 (41.9%)	0.610
Medication			
CP-eq, mg	940.3 (419.9)	957.8 (549.4)	0.794
DZP-eq, mg	6.9 (7.7)	6.3 (9.8)	0.362
Outcome			
Pneumonia	12 (38.7%)	5 (16.1%)	0.046 *

* *p*-value < 0.05; continuous variables are presented as mean (SD); categorical variables are presented as n (%); univariate analysis was performed for each factor between patients who were prescribed MSs and those who were not. Abbreviations: MSs = mood stabilizers; CP = chlorpromazine; DZP = diazepam; eq = equivalent; SD = standard deviation.

**Table 4:** Risk factors for the development of pneumonia after propensity score matching.

	Pneumonia	No Pneumonia	
Variable	(N = 17)	(N = 45)	*p*-Value
Physical Background			
Age, years	66.9 (10.1)	57.0 (11.3)	0.003 *
Sex, male	11 (64.7)	17 (37.8)	0.057
BMI, kg/cm^2^	20.8 (3.3)	20.6 (4.4)	0.711
Comorbidity			
HT, HL, and/or DM	5 (29.4%)	10 (22.2%)	0.555
Medication			
Mood Stabilizers			
Users	12 (70.6%)	19 (42.2%)	0.046 *
LI-eq, mg	355.3 (268.7)	288.6 (471.8)	0.073
VPA Users	10 (58.8%)	9 (20.0%)	0.003 *
Antipsychotic			
Users	17 (100%)	45 (100%)	-
CP-eq, mg	918.8 (534.4)	960.5 (471.0)	0.770
Benzodiazepine			
Users	12 (70.6%)	24 (53.3%)	0.219
DZP-eq, mg	6.8 (6.2)	6.5 (9.4)	0.341

* *p*-value < 0.05; continuous variables are presented as mean (SD); categorical variables are presented as n (%); univariate analysis was performed for each factor between those who developed pneumonia and those who did not. Abbreviations: BMI = body mass index; HT = hypertension; HL = hyperlipidemia; DM = diabetes mellitus; VPA = sodium valproate; LI = lithium carbonate; eq = equivalent; CP = chlorpromazine; DZP = diazepam; SD = standard deviation.

## Data Availability

The data presented in this study are available on request from the corresponding author. The data are not publicly available due to ethical restrictions.
